# Supplementary material for: Comparative multiomics analysis of cell physiological state after culture in a basket bioreactor
Source: Sci Rep. 2022 Nov 23;12:20161. doi: 10.1038/s41598-022-24687-4 (PMC9686226; doi:10.1038/s41598-022-24687-4)

3666059.1:22744570:22745639:+@NW_023666059.1:22744570:22750342:+@NW_023666059.1:22746798:2275

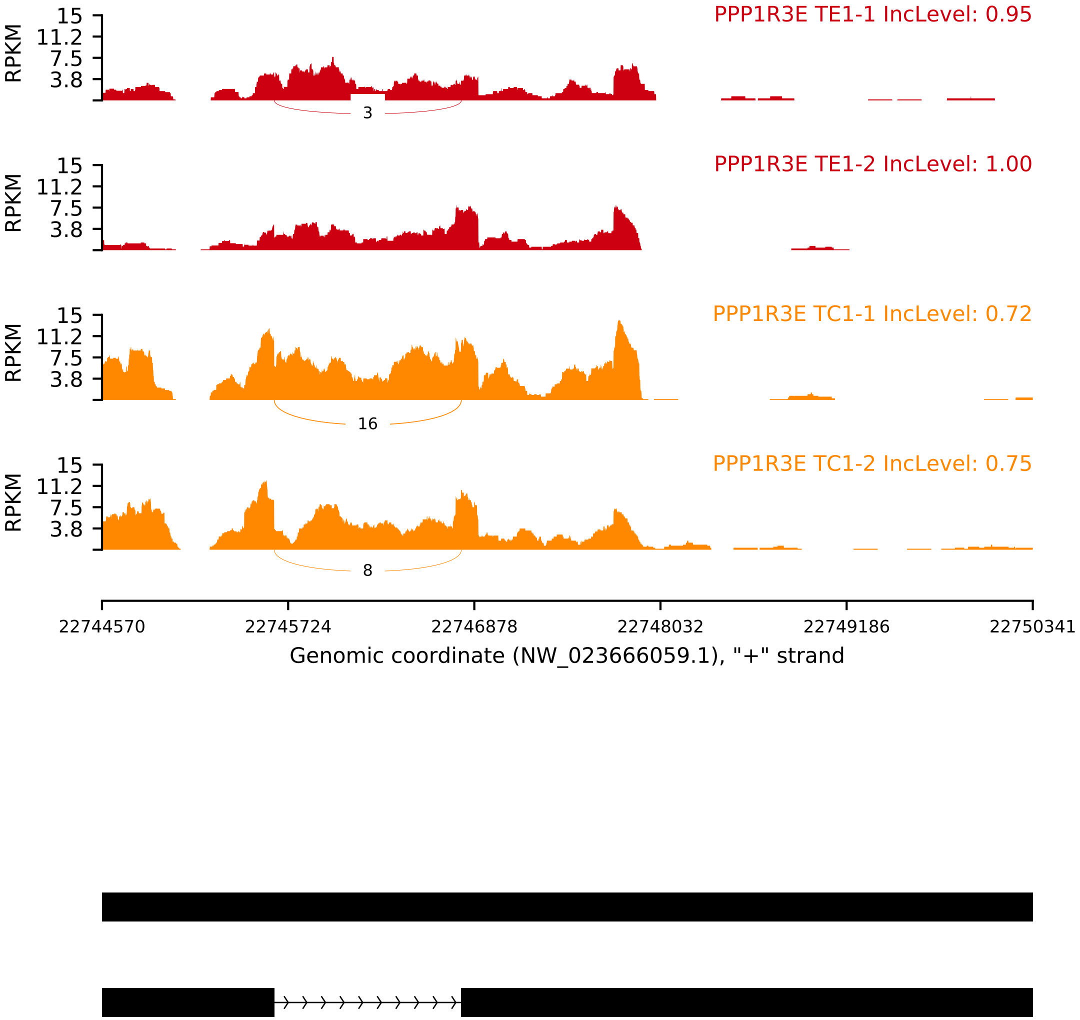

Supplement: Supplementary file 1 — Supplementary Information 1. [file 41598_2022_24687_MOESM1_ESM.zip › raw data/Transcriptomic raw data/7.AS/2.dasplot/TE1vsTC1/RI/12_PPP1R3E_NW_023666059.1_22744570_22745639_+@NW_023666059.1_22744570_22750342_+@NW_023666059.1_22746798_22750342_+.pdf]
